# Chromatolysis: Do injured axons regenerate poorly when ribonucleases attack rough endoplasmic reticulum, ribosomes and RNA?

**DOI:** 10.1002/dneu.22625

**Published:** 2018-08-01

**Authors:** Lawrence David Falcon Moon

**Affiliations:** ^1^ Neurorestoration Group, Wolfson Centre for Age‐Related Diseases, 16‐20 Newcomen Street London SE1 1UL United Kingdom

**Keywords:** chromatolysis, ribonuclease, Angiogenin, endoplasmic reticulum, ribosome

## Abstract

After axonal injury, chromatolysis (fragmentation of Nissl substance) can occur in the soma. Electron microscopy shows that chromatolysis involves fission of the rough endoplasmic reticulum. In CNS neurons (which do not regenerate axons back to their original targets) or in motor neurons or dorsal root ganglion neurons denied axon regeneration (e.g., by transection and ligation), chromatolysis is often accompanied by degranulation (loss of ribosomes from rough endoplasmic reticulum), disaggregation of polyribosomes and degradation of monoribosomes into dust‐like particles. Ribosomes and rough endoplasmic reticulum may also be degraded in autophagic vacuoles by ribophagy and reticulophagy, respectively. In other words, chromatolysis is disruption of parts of the protein synthesis infrastructure. Whereas some neurons may show transient or no chromatolysis, severely injured neurons can remain chromatolytic and never again synthesize normal levels of protein; some may atrophy or die. Ribonuclease(s) might cause the following features of chromatolysis: fragmentation and degranulation of rough endoplasmic reticulum, disaggregation of polyribosomes and degradation of monoribosomes. For example, ribonucleases in the EndoU/PP11 family can modify rough endoplasmic reticulum; many ribonucleases can degrade mRNA causing polyribosomes to unchain and disperse, and they can disassemble monoribosomes; Ribonuclease 5 can control rRNA synthesis and degrade tRNA; Ribonuclease T2 can degrade ribosomes, endoplasmic reticulum and RNA within autophagic vacuoles; and Ribonuclease IRE1α acts as a stress sensor within the endoplasmic reticulum. Regeneration might be improved after axonal injury by protecting the protein synthesis machinery from catabolism; targeting ribonucleases using inhibitors can enhance neurite outgrowth and could be a profitable strategy *in vivo*. © 2018 Wiley Periodicals, Inc. Develop Neurobiol, 2018

## INJURY TO MAMMALIAN AXONS CAN CAUSE a TRANSIENT OR PERSISTENT IMPAIRMENT IN PROTEIN SYNTHESIS

**Figure 1:**
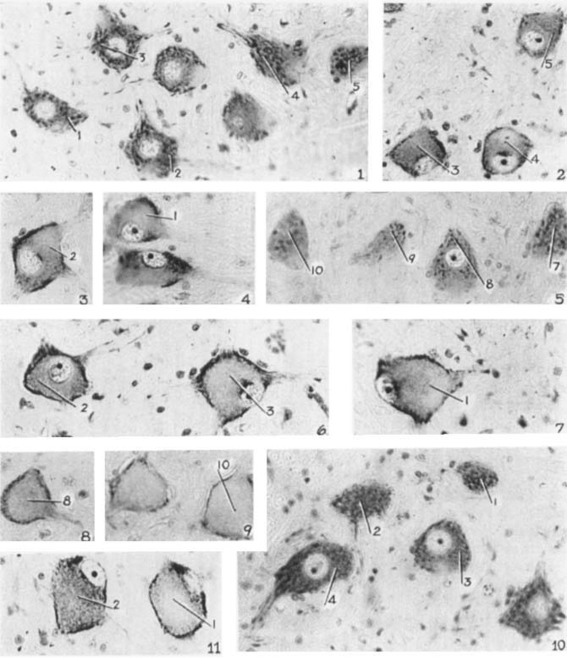
Chromatolysis in neurons involves gross structural abnormality of Nissl substance. Images of toluidine blue‐stained sections of adult monkey cervical spinal cord showing motor neurons after section of a dorsal and ventral root at lumbar or sacral levels. Subpanels 1, 5, and 10 show uninjured neurons. Subpanels 2–5 show sections 3 days after injury and subpanels 6–9 show sections 6 days after injury. Subpanel 11 is 10 days after injury. [Images taken from (Gersh and Bodian, 1943); magnification is X 250].

**Figure 2:**
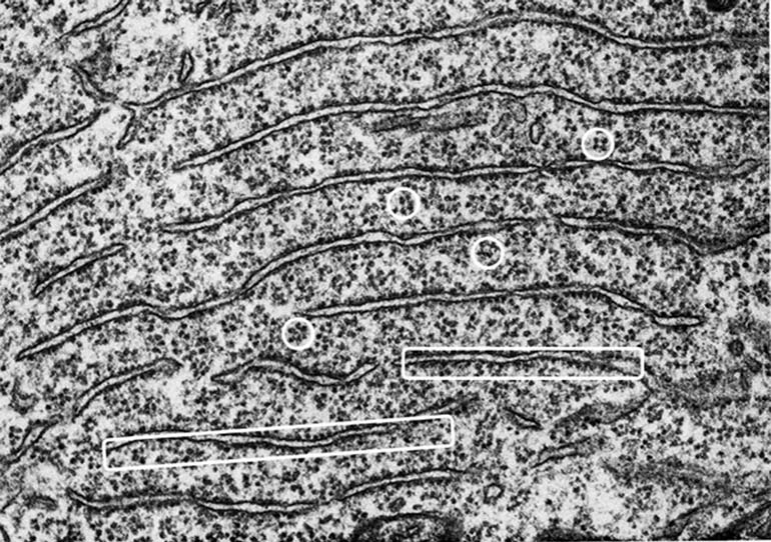
Electron microscopy shows that Nissl bodies in a motor neuron are stacks of rough endoplasmic reticulum whose cisterns are studded externally with ribosomes (white rectangles) and interspersed with rosettes of polyribosomes (white circles). [Image taken from Palay in (Fawcett, 1981) (p.319) in which magnification is not stated but it was noted that fenestrated cisternae are separated by intervals of 0.2 to 0.5 μm].

Why does axonal injury result variably in axon regeneration or collateral sprouting, atrophy or cell death (Thuret et al., 2006)? A long‐standing observation is that after axonal injury, “chromatolysis” occurs in central, autonomic, and peripheral neurons (whether injured peripherally or centrally) (Torvik and Heding, 1969; Lieberman, 1971; Nathaniel and Nathaniel, 1973a, 1973b; Egan et al., 1977a, 1977b; Barron, 1983; Barron, 2004; Kobayashi et al., 2004; Severinsen and Jakobsen, 2009; Johnson and Sears, 2013). This catastrophic event involves dramatic whole‐cell morphological changes that are easily visible under the light microscope (e.g., after cresyl violet or toluidine blue staining for Nissl substance). Its hallmarks are changes in the aggregation, organization and location of “Nissl bodies” as seen under the light microscope (Fig. 1) (Lieberman, 1971). Electron microscopy reveals that Nissl bodies are parallel arrays of cisterns of rough endoplasmic reticulum studded with ribosomes; rosettes of free polyribosomes and monoribosomes are found between the cisterns (Fig. 2) (Matthews and Raisman, 1972; Johnson and Sears, 2013). Each ribosome is a complex of ribosomal RNAs (rRNAs) and proteins that use transfer RNAs (tRNAs) and amino acids to synthesize proteins from mRNAs. In other words, Nissl bodies are a major part of the protein synthesis machinery of a neuron.

**Figure 3:**
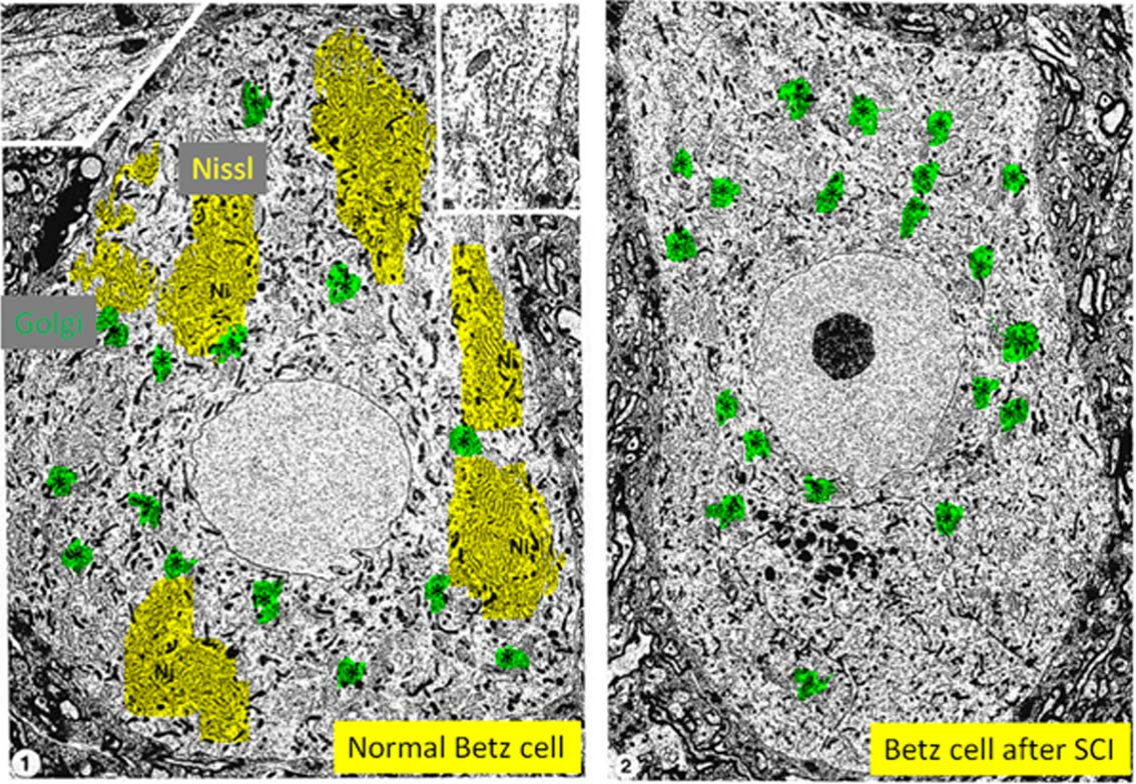
Chromatolysis in CNS neurons involves destruction of the Nissl body component of the protein synthesis machinery. Electron microscope image showing Betz neurons from pericruciate cortex of either (panel 1) a normal adult cat or (panel 2) an adult cat, ten days after spinal cord injury (C2 lateral funiculotomy). Nissl substance is highlighted in yellow (Ni) and aggregates of Golgi are highlighted in green (*). Normal Nissl is no longer visible in the cortical neuron after spinal cord injury. [Images from (Barron and Dentinger, 1979); magnification of panel 1 is X 5,300 (inset is X 21,700) and magnification of panel 2 is X 3,400].

EM shows that chromatolysis is the fragmentation of stacks of rough endoplasmic reticulum leaving clear areas of cytoplasm lacking Nissl bodies; in some cases (see below) this can be accompanied by the disaggregation and/or disassembly of polyribosomes to leave a fine “dust‐like” powder (Cragg, 1970; Matthews and Raisman, 1972; Torvik, 1976; Barron and Dentinger, 1979; Dentinger et al., 1979; Johnson and Sears, 2013). Ribosomes can be depleted from rough endoplasmic reticulum (Lieberman, 1971; Barron, 1989; Baltanas et al., 2011). This can be accompanied by the degradation of monoribosomes (Lieberman, 1971; Engh and Schofield, 1972; Torvik, 1976). Ribosomes and fragments of endoplasmic reticulum can also be found in autophagic vacuoles after axotomy (Matthews and Raisman, 1972; Torvik, 1976) and during Purkinje cell degeneration (Baltanas et al., 2011). The cell body response can also involve dispersion to the soma's periphery of any remaining ribonucleoprotein complexes (Cragg, 1970; Barron and Dentinger, 1979; Dentinger et al., 1979; Johnson and Sears, 2013) (Fig. 3) and movement of the nucleus to an eccentric position. In other words, chromatolysis is the visible disarray of key parts of the protein synthesis machinery (Lieberman, 1971).

Although early investigators regarded chromatolysis as a regressive event, others noted the reversible nature of chromatolysis during successful axon regeneration (Gersh and Bodian, 1943). Some reviews of chromatolysis in the early 1970s concluded that chromatolysis is essential for (and enables) axon regeneration (Cragg, 1970; Torvik, 1976), whereas others proposed that chromatolysis is a catabolic process which can overlap in time with other anabolic processes (Engh and Schofield, 1972; Matthews and Raisman, 1972) such as rRNA synthesis for production of new ribosomes. However, such papers were largely based on data from PNS neurons capable of axon regeneration including spinal motor neurons, dorsal root ganglion (DRG) neurons injured peripherally and sympathetic cervical ganglia (SCG) neurons injured post‐ganglionically (Cragg, 1970; Lieberman, 1971; Watson, 1974). In these neurons, after crush injury (which allows regeneration), levels of total RNA or of newly synthesized total RNA generally increase at most times after injury showing that the anabolic processes generally exceeds the catabolic processes of chromatolysis in regenerating neurons (Watson, 1968; Lieberman, 1971) but not in injured CNS neurons (Barron, 1989). However, the proportion of total RNA that is mRNA is small (typically <5%) compared to rRNA and therefore much of the rise may be dedicated to production of new ribosomes. Indeed, autoradiography and *in situ* hybridization show increases in rRNAs (e.g., 28S rRNA) in motor neurons after peripheral nerve injury [biphasically; see (Kinderman and Jones, 1993; Wells and Vaidya, 1994) and references therein]. In contrast, *in situ* hybridization of DRG neurons undergoing chromatolysis after proteasome inhibition showed dramatic reductions in mRNA in Nissl bodies (Palanca et al., 2014). Furthermore, measuring uptake of radiolabelled protein precursors can inform one about changes in protein synthesis (i.e., from mRNA; rRNA is not translated) after injury (Lieberman, 1971; Barron, 1989). Various experiments show using motor neurons or DRG neurons that transection (which prevents regeneration) rather than crush (which allows regeneration) can cause persistent chromatolysis (Johnson et al., 1985; Johnson et al., 1993) and reduced protein synthesis [(Watson, 1968; Kung, 1971) and see references in (Lieberman, 1974; Torvik, 1976; Barron, 1983)]. For example, large DRG neurons show a ∼50% decrease in protein synthesis between 1 and 35 days after sciatic crush and persistently decreased protein synthesis from 1 to 95 days after sciatic transection (Engh et al., 1971). However, there are counterexamples showing generally increased protein synthesis after crush of regenerating neurons [(Lieberman, 1971) but see (Kung, 1971; Engh and Schofield, 1972; Barron, 1983)].

Data published in the later 1970s and 1980s proved that chromatolysis in (non‐regenerative) CNS neurons involves a reduction in protein synthesis with disassembly and dispersal of the RER (Torvik and Heding, 1969; Barron et al., 1976; Barron et al., 1977; Barron et al., 1982; Barron et al., 1989). Thus, in general, injured, regenerating neurons show increased rates of uptake of radiolabelled amino acids whereas injured CNS neurons show reduced rates of uptake (Barron, 1989). Specifically, CNS neurons undergoing chromatolysis (e.g., after spinal cord injury) show reduced levels of RNA in the nucleus and cytoplasm and reduced protein synthesis per cell within and beyond 24 h (Barron et al., 1976; Barron et al., 1977; Barron et al., 1982; Barron, 1983; Barron, 1989; Barron et al., 1989). This is in marked contrast to spinal motor neurons, SCG or DRG neurons (injured peripherally) which undergo brief chromatolysis that rapidly becomes accompanied by an anabolic phase that reassembles the protein synthesis machinery resulting in axon regeneration (Cragg, 1970; Matthews and Raisman, 1972). Chromatolytic CNS neurons of mammals show disaggregation of free, clustered polyribosomes into single units and degranulation of cisternal membranes whereas other chromatolytic neurons retain clusters of free polyribosomes unless cell death supervenes (Barron, 1983; Barron, 1989). Stressed neurons also form stress granules which may be 100 to 200 nm in size, lack a surrounding membrane and are composed of proteins and RNAs. Stress granules can be a site for degradation of mRNAs or storage of mRNAs until the period of stress has passed (Wolozin, 2012). Not all neurons undergo dramatic chromatolysis after injury (Claman and Bernstein, 1981); this depends on the age of the subject, the type of injury (e.g., crush vs transection), whether the injury is distal from or proximal to the cell body (Goldstein et al., 1987) and, possibly, whether there are spared collaterals proximal to the injury site (Lieberman, 1974; Barron, 2004).

In the 2000s and 2010s, transcriptomic or proteomic experiments have not usually reported a global suppression of protein synthesis in homogenates of neurons and glia after PNS crush although the expression level of many transcripts and proteins do go down. It may be relevant that many of these experiments have been normalised in a way that might mask overall changes in protein synthesis (e.g., RNA sequencing experiments are often normalised to the FPKM; number of fragments per kilobase million). However, key gene changes are generally corroborated by cell‐type specific *in situ* hybridization data or qRTPCR data normalised to an “invariant” RNA (*n.b*., 28S rRNA changes its level biphasically in DRG after nerve injury [(Kinderman and Jones, 1993); see also (Wells and Vaidya, 1989, 1994)]. Nonetheless, in the 2020s, it will be useful to use single‐cell‐type methods to determine which, if any, injuries induce global reductions in protein synthesis during phases of chromatolysis on a per‐neuron basis. New state‐of‐the‐art methods exist for studying protein synthesis (Iwasaki and Ingolia, 2017). Again, it is important to emphasize that not all injuries cause chromatolysis, and that the catabolic consequences of chromatolysis may not cause an overall (net) loss in protein synthesis capacity if the anabolic response is quick and strong: considering all the available data, protein synthesis appears to increase in neurons that sprout or regenerate.

In conclusion, given the ultrastructural hallmarks of chromatolysis (e.g., fission or dispersal of the RER and disaggregation of polyribosomes), the most plausible explanation is that the functional consequence of chromatolysis is disruption of protein synthesis which can be transient or permanent depending on a variety of factors including the type and the location of injury.

## DO RIBONUCLEASES CAUSE FRAGMENTATION, DISPERSAL AND DEGRANULATION OF ROUGH ENDOPLASMIC RETICULUM, DISSOCIATION OF POLYRIBOSOMES AND DEGRADATION OF MONORIBOSOMES AND RNA AFTER INJURY?

**Figure 4:**
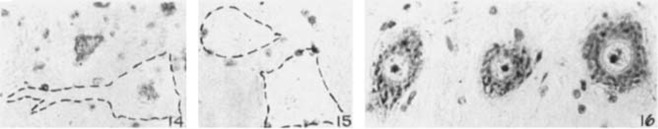
Nissl substance (i.e., rough ER) can be destroyed with ribonuclease. L7 spinal cord neurons stained for Nissl using toluidine blue either without (subpanels 14, 15) or with (subpanel 16) treatment of fixed tissue sections with ribonuclease. The cell bodies are outlined with broken lines. Nissl bodies are visible as dark patches in the cytoplasm in subpanel 16 but are not visible in subpanels 14 or 15. Nucleolar basophilic staining is also nearly abolished. Staining of chromatin of glia is not affected. [Image taken from (Gersh and Bodian, 1943); magnification is X 250].

Perhaps amazingly, given that chromatolysis was first reported in the late 1800s [by Nissl in 1892 and Marinesco in 1898; (Severinsen and Jakobsen, 2009)], it is not yet known what molecule(s) fragment and degranulate rough endoplasmic reticulum, disaggregate polyribosomes and degrade monoribosomes although the involvement of ribonucleases is plausible (Table 1). In 1943, Gersh and Bodian proved (using light microscopy) that Nissl substance in spinal motor neurons contains RNA by showing that treatment of fixed spinal cord sections with ribonuclease entirely abolished subsequent Nissl staining (Fig. 4). They went further and suggested that chromatolysis might occur due to the activity of ribonucleases *in vivo*. In the 1960s, it was shown that chromatolysis in injured facial nerve neurons requires new protein synthesis (Torvik and Heding, 1969) and the authors wondered whether an enzyme might be responsible for dispersion of the Nissl substance (Torvik and Heding, 1969).

**Table 1:** Chromatolysis Involves a Set of Events that Might be Caused by One or More Ribonucleases

Cellular event	Examples of candidate ribonucleases
Degradation of mRNA	RNase 1 (Saxena et al., 1992) RNase 2 (Saxena et al., 1992) Polysome‐Bound Endonuclease (PMR1) (Schoenberg and Maquat, 2012) GTPase‐activating protein binding protein (G3BP‐1) (Schoenberg and Maquat, 2012) IRE1α (Li et al., 2013)
Degradation of rRNA	Ribonuclease T2 (Haud et al., 2011)
Degradation of tRNA into tiRNA	RNase 5 (Saxena et al., 1992; Pizzo et al., 2013)
Fragmentation/fission/fusion of rough endoplasmic reticulum	RNase 1 can cause dose‐dependent changes in endoplasmic reticulum whilst EndoU/PP11 ribonucleases can dynamically regulate smooth and rough endoplasmic reticulum (Schwarz and Blower, 2014)
Degranulation of rough endoplasmic reticulum	Not known
Degradation of rough endoplasmic reticulum	RNase T2 (Haud et al., 2011)
Disaggregation of polyribosomes into monoribosomes	Ribonuclease 1 (Gerashchenko and Gladyshev, 2017)
Degradation of monoribosomes	RNase T2 (Haud et al., 2011) and Ribonuclease 1 (Gerashchenko and Gladyshev, 2017)
Promotion of rRNA transcription	RNase 5
Stress sensor	IRE1α (Li et al., 2013)

Almost nothing is known about the role of these ribonucleases in chromatolysis in neurons; rather, the evidence for these ribonucleases playing a role in these cellular events is drawn from what is known from other cell types in normal or stressed situations.

In 1970, Cragg asked “What is the signal for chromatolysis?” and he considered various hypotheses including signals conveyed by retrograde transport from the site of injury. However, he concluded “The hypothesis that the neurone produces a substance that represses neuronal RNA production, and loses some of this repressor when the axon is injured or when it sprouts, comes nearest to explaining the experimental findings as they are known at present”. With the benefit of hindsight, this conclusion might explain the anabolic response seen in chromatolytic neurons that regenerate, but it cannot explain the catabolic response seen in chromatolytic CNS neurons that were described after 1970. Since Cragg's review, many groups have described retrograde signals from injury sites that can induce neuronal cell body responses (Hanz et al., 2003; Hanz and Fainzilber, 2006; Rishal and Fainzilber, 2014; Ying et al., 2014; Hu, 2016). Here, I consider the possibility that the catabolic response is executed in part by various ribonucleases (Table 1); the anabolic mechanism whereby the protein synthesis machinery is built or rebuilt is discussed briefly at the end of this review.

From the 1970s, there are beautiful ultrastructural images of sympathetic ganglia undergoing chromatolysis after postganglionic injury (Matthews and Raisman, 1972). Biochemical experiments in the 1980s then revealed that uninjured sympathetic ganglia contain inactive ribonucleases [see references in (Bates et al., 1985b)] and that the total activity levels of alkaline ribonucleases increase in sympathetic ganglia after postganglionic nerve injury: this is the result of increased synthesis of ribonucleases as well as activation of existing ribonucleases. This activity becomes progressively restrained by one or more endogenous Ribonuclease Inhibitors (Bates et al., 1987, 1985a, 1985b). This is consistent with the idea that chromatolysis in sympathetic ganglia is due to ribonuclease activity and that any anabolic response occurs after progressive inhibition of cytoplasmic ribonucleases. [An increase in nuclear ribonuclease activity may be required for processing of newly synthesized RNA in the anabolic phase (Bates et al., 1987; Pizzo et al., 2013)]. To my knowledge, these ribonucleases and ribonuclease inhibitors have not been studied to any great extent in DRG neurons or CNS neurons.

In the 1980s it was suggested that some “suicide enzyme”, perhaps a “powerful ribonuclease”, is responsible for disassembly of polyribosomes into dust‐like particles in a developing chick CNS nucleus (*nucleus magnocellularis*) deprived of all afferent input (Rubel et al., 1991). [Because RER was not seen to be degraded in this study, other mechanisms may be responsible for this phenomenon; see below]. Polyribosomes are also turned to dust in severely injured chromatolytic sympathetic ganglia (Matthews and Raisman, 1972) and in dendrites of uninjured dentate gyrus neurons after treatment of fixed hippocampal tissue blocks with ribonuclease of “type II” (Sigma) (Steward, 1983). Indeed, because polyribosomes are linked together by mRNA, ribonuclease treatment can degrade mRNA and dissociate them [(Warner et al., 1963; Gerashchenko and Gladyshev, 2017) and see p. 305 in (Fawcett, 1981)].

If ribonucleases can disassociate polyribosomes into monoribosomes, what causes degradation of monoribosomes? Every ribosome is made of two subunits each composed of a complex of rRNAs with proteins; indeed, rRNA comprises the predominant material by weight (comprising ∼60% of the ribosome mass). It is not surprising, therefore, that exogenous treatment using various ribonucleases can degrade monoribosomes into “dust‐like” fragments *in vitro* (Steward, 1983; Rubel et al., 1991) [see also (Blasi et al., 2000; Gerashchenko and Gladyshev, 2017)]. Interestingly, endogenous ribonucleases accompany ribosomes (Bransgrove and Cosquer, 1978; Bates et al., 1985a; Bates et al., 1987; Schoenberg and Maquat, 2012) including in the adult mammalian brain (Datta and Ghosh, 1962) presumably constrained by an endogenous inhibitor (Allam et al., 2017). As will be seen next, particular ribonucleases are plausibly responsible for fragmentation of rough endoplasmic reticulum, dissociation of polyribosomes, degradation of monoribosomes and decay of RNA (Table 1).

## WHICH RIBONUCLEASE(s) MIGHT CAUSE FRAGMENTATION OF ROUGH ENDOPLASMIC RETICULUM, DISASSOCIATION OF POLYRIBOSOMES, DEGRADATION OF RNA, AND MONORIBOSOMES AFTER AXOTOMY?

### Which Ribonuclease(s) Might Degrade RNAs during Chromatolysis?

A variety of ribonucleases might degrade RNAs during chromatolysis such as those in the secreted, vertebrate ribonuclease family (Ivanov and Anderson, 2011; Nicholson, 2011) whose canonical member is bovine pancreatic RNase A (often known as Ribonuclease 1 or pancreatic RNase). In humans there are eight canonical Ribonucleases in this family. RNases have different specificities and may play different roles after neuronal injury. For example, Ribonuclease 5 cleaves tRNA (but not mRNA or rRNA) whereas others including Ribonucleases 1 and 2 cleave mRNA, rRNA, and tRNA (Saxena et al., 1992) (Table 1).

Where might these ribonucleases come from? There is one report that chromatolysis can be prevented by protein synthesis inhibitors (Torvik and Heding, 1969) indicating that chromatolysis may be due to a newly‐synthesized enzyme (Bates et al., 1985a; Mami et al., 2016). Alternatively, perhaps ribonucleases that are associated with ribosomes (Datta and Ghosh, 1962; Bransgrove and Cosquer, 1978; Bates et al., 1985a; Bates et al., 1987; Schoenberg and Maquat, 2012) become activated after injury (Allam et al., 2017). Ribonucleases are also known to change their subcellular distribution after injury or during stress (Ivanov and Anderson, 2011; Nicholson, 2011). Data obtained using non‐neural cells show that under conditions of stress, Ribonuclease 5 moves to the cytoplasm including to stress granules where it becomes activated and hemisects tRNAs into tiRNAs: this impairs translation of many proteins, although some proteins essential for cell survival continue to be manufactured (Pizzo et al., 2013). A normally nucleolar ribonuclease, B23/Nucleophosmin, appears to be present at higher levels in the cytoplasm of chromatolytic neurons (Baltanas et al., 2011) where it might contribute to dramatic loss of mRNA (Palanca et al., 2014). Finally, perhaps ribonucleases are taken up from the extracellular environment: many ribonucleases are also secreted and are found in cerebrospinal fluid (CSF) (Yasuda et al., 1993) including Ribonuclease 2, Ribonuclease 3 (Eosinophil Cationic Protein; ECP), and Ribonuclease 5 (also known as Angiogenin) (Ng et al., 2011) and increased levels are found in CSF and blood after spinal cord injury in humans including Ribonuclease 5 (Rabin et al., 1977; Ng et al., 2011). Several (but not all) ribonucleases cause neuronal injury when given intrathecally including Ribonucleases 2 and 3 (Newton et al., 1994). Indeed, Ribonuclease 2 is also known as eosinophilic derived neurotoxin; it causes rapid neuronal cell death when it is injected intrathecally (Sorrentino et al., 1992; Newton et al., 1994). Injection of various Ribonucleases, including 1 and 5, into cells results in the degradation of the cells' RNA and causes cell death (Saxena et al., 1991; Saxena et al., 1992). Thus, it is possible that chromatolysis may be due to uptake of a ribonuclease from the extracellular environment after injury; however, this would need to be reconciled with the fact that chromatolysis tends to start centrally, sometimes (but not always) with sparing of peripheral rims of Nissl (Barron, 2004).

### Which Ribonuclease(s) Might Cause Fragmentation of Rough Endoplasmic Reticulum?

It is not known what causes fragmentation or disarray of rough endoplasmic reticulum in chromatolytic neurons. However, extensive and rapid fission of endoplasmic reticulum in CNS dendrites can be triggered by increases in intracellular calcium *in vitro* and in adult cortical neurons during global ischemia *in vivo* (Kucharz et al., 2009; Kucharz et al., 2011, 2013; Zhao and Blackstone, 2014). Interestingly, this process is reversible: fusion of fragments occurs if the fissile stimulus (e.g., K^+^) is washed out or if an NMDA receptor antagonist is applied (Kucharz et al., 2013). It is not yet known whether smooth endoplasmic reticulum in the axon or rough endoplasmic reticulum in the cell body also undergoes fission under these circumstances (Kucharz et al., 2013).

The mechanism(s) by which endoplasmic reticulum is fragmented is not known but there is evidence from other cell types that calcium‐dependent ribonucleases in the EndoU/PP11 family dynamically regulate endoplasmic reticulum (Zhao and Blackstone, 2014). In *Xenopus* oocytes XendoU is bound to the endoplasmic reticulum where it can degrade RNA and remove ribosomes and ribonucleoproteins. This causes expansion of rough endoplasmic reticulum at the expense of smooth endoplasmic reticulum: depletion of XendoU caused an expansion of rough endoplasmic reticulum sheets at the expense of smooth endoplasmic reticulum tubules which could be rescued by XendoU in a ribonuclease‐dependent manner (Schwarz and Blower, 2014). However, it is not clear whether EndoU/PP11 family members cause fragmentation *per se* rather than switching of ER type from rough to smooth by degranulation. Ribonuclease 1 can also cause dose‐dependent changes in endoplasmic reticulum in non‐neural cells (Schwarz and Blower, 2014). Interestingly, there is one report of depletion of rough endoplasmic reticulum with expansion of smooth endoplasmic reticulum in injured adult cat red nucleus neurons after spinal cord injury (Barron et al., 1975) but increases in smooth endoplasmic reticulum after injury to other neurons has not been reported more widely (Barron, 1983). It will be important to determine whether calcium‐dependent ribonucleases cause degranulation and/or fragmentation or depletion of rough endoplasmic reticulum in neuronal cell bodies.

### Which Ribonuclease(s) Might Cause Disassociation of Free Polyribosomes or Degradation of Monoribosomes?

Treatment of purified ribosomes (from mouse liver) with Ribonuclease 1 causes disassembly of polyribosomes (into monoribosomes) and degradation of monoribosomes *in vitro* whereas treatment with other ribonucleases (e.g., T1) only causes disassembly of polyribosomes into monoribosomes and does not cause degradation of monoribosomes into fragments (Gerashchenko and Gladyshev, 2017) [*n.b*., T1 is a fungal ribonuclease so some other ribonuclease would have to be responsible in mammals (Blasi et al., 2000)].

Cellular stress leads to formation of stress granules in which mRNAs may be stored or degraded (Wolozin, 2012). Stress also causes “No‐go decay” and “Nonsense mediated decay” of mRNAs by ribonucleases that release ribosomes from endoplasmic reticulum (Schoenberg and Maquat, 2012). Stress granules may contain ribonucleases that can cleave mRNAs, including Polysome‐Bound Endonuclease (PMR1) and GTPase‐activating protein binding protein (G3BP‐1), or that can cleave tRNAs including Ribonuclease 5 (Schoenberg and Maquat, 2012). Stress granules can also be induced in an eIF2α‐independent manner indirectly by Ribonuclease 5 (Emara et al., 2010). Heretofore, these ribonucleases have not been studied much in axotomised neurons.

The role of ribonucleases in chromatolysis after axonal injury could be explored by overexpressing those thought to be protective and by using conditional knockout methods to deplete those thought to be deleterious (and *vice versa*). For example, any floxed Ribonuclease gene could be deleted in sensory neurons expressing Advillin‐Cre^ERT2^ upon application of tamoxifen (Lau et al., 2011). It might also be possible to restrict the subcellular localization of specific RNases (e.g., by adding nuclear localization signals).

## WHY DON'T ENDOGENOUS, POTENT RIBONUCLEASES CONSTITUTIVELY CAUSE CHROMATOLYSIS IN UNINJURED NEURONS?

A variety of mechanisms constrain the activity of ribonucleases. In the intact brain and in uninjured SCG, net ribonuclease activity is low due to a surplus of endogenous inhibitors of ribonucleases (Burton et al., 1980; Bates et al., 1985b). Other ribonucleases require activation by phosphorylation (e.g., PMR1), by oligomerization (e.g., IRE1α), or by calcium entry (e.g., XendoU) which happens after axotomy or during ER stress (Schoenberg and Maquat, 2012). The secreted vertebrate family of ribonucleases is normally tightly controlled by the mammalian Ribonuclease Inhibitor 1 (RNH1) (Dickson et al., 2005). RNH1 is found primarily in the cytoplasm, where it binds to these RNases with remarkably high affinities (Dickson et al., 2005) and inhibits their activities, although it can also be found in the nucleus, in mitochondria, in stress granules (Furia et al., 2011) and associated with ribosomes (Allam et al., 2017). Cytoplasmic inhibition of ribonucleases is disrupted in cellular stress situations, e.g., by oxidative stress (Dickson et al., 2005): net activity of ribonucleases is increased transiently in SCG during chromatolysis (Bates et al., 1985a). Unfettered ribonucleases might then degrade polyribosomes (into free monoribosomes) and monoribosomes (into ribosome fragments). Indeed, RNH1 deficiency leads to decreased polyribosome formation whereas overexpression of RNH1 promotes polyribosome formation (Allam et al., 2017). During stress in non‐neuronal cells, RNH1 is translocated to the nucleus and Ribonuclease 5 is exported from the nucleus (Pizzo et al., 2013). This results in a reduction of rRNA production in the nucleolus and simultaneous break‐down of tRNAs in the cytoplasm. If ribonuclease activity remains increased persistently in CNS or PNS neurons, then this could help explain atrophy and failure of long‐distance axon regeneration after proximal CNS injury.

## CHROMATOLYSIS CAN INVOLVE RIBOPHAGY AND RETICULOPHAGY

Work in the late 1960s and early 1970s showed evidence of fragmentation of RER and degradation of RER, polyribosomes and monoribosomes in autophagic vacuoles and dense bodies of severely chromatolytic neurons (Dixon, 1967; Matthews and Raisman, 1972) perhaps formed from membranes of fragments of RER itself (Matthews and Raisman, 1972). Later, in the 1990s, it was shown that one can have complete destruction of polyribosomes into dust without destruction of RER (as seen after de‐afferentation of the *nucleus magnocellularis* in developing chick (Rubel et al., 1991)). There is recent data (from non‐neural cells) showing that RNA, protein and membrane components of ribosomes and endoplasmic reticulum can be degraded by different mechanisms called ribophagy and reticulophagy, respectively (Kraft et al., 2008; Cebollero et al., 2012) in acidic lysosomes that contain numerous hydrolytic enzymes. Ribonuclease T2 may degrade rRNA and ribosomes in lysosomes during ribophagy: it is the only ribonuclease active at acidic pH and mutations in this ribonuclease cause a lysosomal storage disorder in neurons in humans and fish (Haud et al., 2011). Accordingly, Ribonuclease T2 may play a role in phagocytosis of ribosomes and endoplasmic reticulum in autophagic vacuoles during severe chromatolysis (Matthews and Raisman, 1972).

The mechanism of bulk autophagy in the nervous system can involve Autophagy‐related proteins (Atg) including Atg5 and Atg7 (Hara et al., 2006; Komatsu et al., 2006). The molecular mechanisms of these forms of autophagy are beginning to be explored and can involve Atg1 and Atg7 (Cebollero et al., 2012). A Ubiquitin protease (Ubp3) and its cofactor (Bre3) are involved in ribophagy (but not bulk autophagy) (Kraft et al., 2008). With respect to reticulophagy, Atg‐related proteins involved in this process appear to be regulated downstream of the ER stress sensors IRE1α, ATF6, and PERK (Cebollero et al., 2012). ER stress can induce autophagy in other cell types and it is possible that axonal or somatic ER stress is the initiator of chromatolysis (Ying et al., 2014). For example, activation of the IRE1α‐XBP pathway indirectly leads to synthesis of Ribonuclease 5 [after kidney injury; (Mami et al., 2016)]. Moreover, IRE1α is a ribonuclease that is known to degrade a wide range of mRNAs in the ER in a process known as RIDD [regulated IRE1α‐dependent decay; (Li et al., 2013)]. The mechanisms of ribophagy and reticulophagy and their relationship to ER stress and chromatolysis need to be investigated in neurons in more detail; it is likely that these terms (i.e., stress, autophagy, chromatolysis) mean different things to different researchers and that clear definitions will be required to avoid confusion when trying to understand to what extent these processes overlap, cause one another, run in parallel or interact.

## DOES AN AXON FAIL TO REGENERATE WHEN ITS RNAS AND RIBOSOMES ARE DEGRADED LOCALLY?

Even if an injured neuron does not become chromatolytic it is conceivable that axonal RNA and ribosomes are degraded by ribonucleases. PNS and some CNS axons synthesize proteins in their axons (Verma et al., 2005; Twiss and Fainzilber, 2009; Rishal and Fainzilber, 2014) and axonal RNAs can be translated locally after injury (Gumy et al., 2010); some serve as a retrograde injury signal (Twiss and Fainzilber, 2009). Axonal ribosomes often (but not always) eluded detection in the electron microscope (Bunge, 1973; Twiss and Fainzilber, 2009; Gold et al., 2017): is it also possible that injury causes cleavage of RNA and/or ribosomes in axons? Local degradation of RNA and ribosomes within an injured branch might help explain why one branch of an axon fails to regenerate whereas other zones sprout collaterals, perhaps co‐ordinated by mitochondria with ribosomes (Spillane et al., 2013; Gold et al., 2017). There is some evidence that IRE1α ribonuclease is active in neuronal processes (Hayashi et al., 2007) but in general little is known about the activity of ribonucleases in injured neurons.

## MIGHT ATTENUATION OF CHROMATOLYSIS BE THERAPEUTICALLY BENEFICIAL?

The mechanisms whereby chromatolysis is attenuated after axotomy above are not well understood but in non‐neural cells, if cellular stress subsides and growth resumes, Ribonuclease 5 returns from the cytoplasm to the nucleus where it stimulates rRNA transcription (Pizzo et al., 2013). The anabolic phase in neurons includes synthesis of new rRNA, mRNA and tRNA (Kinderman and Jones, 1993; Wells and Vaidya, 1994) and may include salvage of RNAs from stress granules and conversion of smooth ER back into rough ER by fusion. Biogenesis of ribosomes is a complicated matter; the interested reader is referred to a short review (Olson and Dundr, 2015) or book on the nucleolus in which ribosome biogenesis in uninjured or stressed cells is described (Olson, 2011). Compensatory responses to chromatolysis and loss of cytoplasmic RNA after proteasome inhibition in DRG neurons can include: an increase in the size and number of nucleoli per neuron; sustained nucleolar transcription; increased rRNA synthesis; and upregulation of some mRNAs including B23 ribonuclease involved in ribosome biogenesis (Palanca et al., 2014; Riancho et al., 2014). However, little else is known about the mechanisms by which chromatolysis is reversed in neurons.

Interestingly, injured spinal cord neurons upregulate the rat Ribonuclease Inhibitor (RNH1, also known as SCIRR39) *in vitro* and *in vivo* (Zhao et al., 2014) and, in PC12 cells, overexpression of RNH1 enhances neurite outgrowth whilst knockdown of RNH1 reduces neurite outgrowth (Zhao et al., 2013). It is plausible therefore that RNH1 promotes neurite outgrowth by inhibiting ribonucleases although other mechanisms could be responsible [such as binding of PTEN (Kim et al., 2011)]. It is also not known if manipulation of RNH1 modifies chromatolysis in neurons and more remains to be done to determine whether ribonucleases constrain growth in axons.

Targeting the ER stress response might modify chromatolysis; there are reports it can increase or decrease recovery after PNS or CNS injury including spinal cord injury (Penas et al., 2011; Li et al., 2013; Hetz and Mollereau, 2014; Onate et al., 2016). Future work may show if targeting the ER stress response can reduce fragmentation of rough endoplasmic reticulum or protect ribosomes and RNA in neurons. Although chromatolysis may have evolved as a mechanism to allow neuronal survival after injury (Palanca et al., 2014; Riancho et al., 2014), it may not be an optimal solution with respect to axon regeneration and with modern molecular therapies it might be possible both to maintain cell survival and accelerate the onset of axon regeneration before other factors (e.g., scar formation) intervene.

Neurons that do extend axons effectively after injury express a cohort of key “Regeneration‐Associated Genes” (RAGs) and maintain low levels of key Regeneration‐Inhibiting Genes (RIGs) (Chandran et al., 2016). In contrast, injured CNS neurons often fail to produce adequate levels of proteins from many RAGs (Tetzlaff et al., 1991). This contributes to their regenerative failure as does the fact that CNS axon growth is restricted by cavity formation and growth‐inhibitory extracellular substances including myelin‐associated glycoprotein and chondroitin sulfate proteoglycans. To date, many methods for promoting PNS or CNS axon regeneration have focused manipulation of one or a small number of genes. Some strategies have achieved regeneration of axons in the PNS and CNS by overexpressing a single RAG (e.g., KLF7; (Moore et al., 2009)) or reducing levels of a RIG (e.g., PTEN; (Jin et al., 2015)).

However, some severely injured neurons undergo persistent chromatolysis and atrophy. Perhaps in severely injured neurons, this strategy of overexpressing one or a small number of genes is unlikely to succeed unless those genes can prevent the collapse of (or induce the restitution of) much of the protein synthesis machinery. Might this be feasible? Chromatolysis generally takes a few days to reach a maximum even when injury is within a few millimeters of the cell body (Matthews and Raisman, 1972). This indicates that early intervention after injury may be possible to prevent collapse of this part of the protein synthesis machinery which may lead to a more favorable outcome. Alternatively, ribonucleases might cause irreversible cleavage of RNA and ribosomes within hours of injury but the diffusion or dispersion of Nissl substance might take longer. Chromatolytic neurons often revert to a more‐normal phenotype if they regenerate (Matthews and Raisman, 1972; Johnson and Sears, 2013). In cultured neurons and organotypic slices, fission of endoplasmic reticulum in dendrites can be followed by fusion (i.e., it is reversible): it does not affect neuronal survival (Kucharz et al., 2009; Kucharz et al., 2013). Chromatolysis is also reversible in CNS neurons. For example, after thoracic rubrospinal tract injury, red nucleus neurons undergo mild chromatolysis that is reversed with time whereas this is largely not the case after cervical rubrospinal tract injury (Egan et al., 1977b). Various treatments have also been shown to prevent or reverse atrophy in CNS neurons including neurotrophin treatment (Kobayashi et al., 1997; Kwon et al., 2007) and chondroitinase ABC (Carter et al., 2008; Carter et al., 2011) even after long delays (Kwon et al., 2002). Empirical evidence is needed to determine whether blocking the chromatolytic response leads to cell death or whether it can accelerate axon regeneration. Downregulation, subcellular compartmental sequestration, inhibition or neutralization of ribonucleases may be ways to achieve this.

In conclusion, ribonucleases may contribute to chromatolysis, ER stress, ribophagy and reticulophagy after neuronal injury. Identification of which ribonucleases play deleterious role and which ribonucleases play pro‐regenerative roles could be an important step in developing new therapies for repair of nervous system injuries.

This work was supported by a grant from the Wings for Life foundation and through a grant to the “AxonRepair” consortium from ERA‐NET NEURON that is co‐sponsored by the Medical Research Council. Thanks to Emeritus Professor Thomas Sears and Professors Mike Fainzilber and Simone Di Giovanni for providing feedback on a draft.
